# Association between plasma homocysteine and myocardial SPECT abnormalities in patients referred for suspected myocardial ischaemia

**DOI:** 10.5830/CVJA-2011-048

**Published:** 2012-07

**Authors:** Alfred Ankrah, John Buscombe, Mike Machaba Sathekge

**Affiliations:** Department of Nuclear Medicine, University of Pretoria and Steve Biko Academic Hospital, Pretoria, South Africa; Department of Nuclear Medicine, University of Pretoria and Steve Biko Academic Hospital, Pretoria, South Africa; Department of Nuclear Medicine, University of Pretoria and Steve Biko Academic Hospital, Pretoria, South Africa

**Keywords:** plasma homocysteine, coronary artery disease, myocardial SPECT indices, myocardial ischaemia

## Abstract

**Background:**

Elevated plasma homocysteine level has emerged as a relatively newly recognised risk factor for coronary artery disease (CAD). However, reduction of plasma homocysteine levels in large prospective studies did not appear to reduce the risk for subsequent cardiac events. In this study, we investigated the association between plasma homocysteine levels and quantitative indices of myocardial perfusion SPECT imaging in patients referred for myocardial ischaemia.

**Methods:**

Quantitative myocardial perfusion SPECT indices were obtained for 120 patients who were recruited for the study. All patients underwent a two-day rest–stress myocardial perfusion imaging. Plasma venous sampling was done on all patients after an overnight fast. Of the 120 participants (mean age 56 years, 53% males), 33% had elevated plasma homocysteine levels. The plasma homocysteine level was then compared to the results of imaging and other known risk factors.

**Results:**

After adjustment for traditional risk factors of coronary artery disease, patients with elevated homocysteine levels had a significantly higher mean summed stress score (SSS) (11.3 vs 6.9, *p* = 0.02) than patients with a normal homocysteine level. This was true for both single- and multivessel disease. Also, patients with elevated homocysteine levels had a higher stress end-systolic volume (SESV) (137 vs 105 ml, *p* = 0.03) and lower post-stress left ventricular ejection fraction (SEF) (54 vs 64%, *p* = 0.02). The patients with elevated plasma homocysteine levels also had a significantly lower mean body mass index (BMI) (26.6 vs 30.6 kg/m^2^, *p* = 0.002). There was a significant relationship between the total number of known risk factors in a patient with CAD and the proportion of patients presenting with elevated plasma homocysteine levels (*p* = 0.03). Also, the extent of infarct, as measured by the summed rest score (SRS), was more closely correlated with an elevated homocysteine level than with the degree of ischaemia.

**Conclusion:**

There was a correlation between plasma homocysteine level and the presence and extent of myocardial perfusion abnormalities in patients with established coronary artery disease, in particular those with multiple risk factors and multi-vessel infarction.

## Abstract

In the past two decades, plasma homocysteine level has been extensively investigated and proposed as an independent cardiovascular disease risk factor.^1^ An elevated homocysteine level was first shown to be a risk factor for coronary artery disease (CAD) and later an independent risk factor for thromboembolism.^2^ Elevated homocysteine level has been linked in many studies to greater risk of adverse cardiovascular disease outcomes, which include myocardial infarction, stroke and cardiovascular mortality.^3^-^7^

However, clinical trials have not been able to demonstrate a reduction in clinical cardiovascular endpoints after therapeutic reduction of plasma homocysteine levels.^8^ This is in contrast to the outcome with other traditional risk factors of CAD, such as lowering plasma cholesterol levels. This is at variance with animal studies, which have shown that elevated homocysteine level is associated with increased cardiac dysfunction, and that lowering plasma homocysteine levels with folic acid may have a beneficial effect.^9^ The relationship between homocysteine level and myocardial dysfunction, and the potential therapeutic usefulness of lowering plasma homocysteine level is still being explored.

Myocardial perfusion SPECT is a validated imaging technique providing for both diagnosis and risk stratification of the possibility of future cardiac events.^10^ Validated automated algorithms provide semi-quantitative assessment of myocardial perfusion and left ventricular systolic function.^11^ In this study we aimed to assess the correlation between homocysteine level and myocardial SPECT abnormalities in 120 patients with suspected myocardial ischaemia.

## Methods

This prospective, open-label trial, approved by the ethics committee of the University of Pretoria, Faculty of Health Science, was designed to evaluate the association between homocysteine level and myocardial SPECT abnormalities. A total of 120 patients referred to our department with suspected myocardial ischaemia for myocardial perfusion imaging were recruited, after giving informed consent.

Information was obtained from each patient concerning any history of hypertension, diabetes mellitus, smoking and dyslipidaemia. Each patient’s weight and height were recorded. Exclusion criteria included pregnancy, use of vitamin supplementation (for more than five days a week in the previous three months), and renal insufficiency (defined as creatinine level > 20 mmol/l). Patients with an acute coronary syndrome of less than 48 hours and those unable to undertake a stress study because they were unfit were also excluded.

## Measurement of homocysteine

Prior to stressing, venous blood was obtained from the fasting patients and immediately sent to the laboratory where it was centrifuged and frozen. Total homocysteine level was determined by enzymatic assay and by Abbott florescence polarisation immunoassay. This method has been shown to correlate well with both gas chromatography–mass spectrometry and high-performance liquid chromatography (HPLC) methods.^12^

Elevated homocysteine level was defined as plasma level > 12 μmol/l. The homocysteine results were not known at the time of analysis of the myocardial perfusion scintigraphy.

## Stress protocol

All patients were imaged using the department’s standard two-day ^99m^Tc MIBI protocol. Patients were stressed physically on a bicycle ergometer or with pharmacological agents. Pharmacological stress was performed using an infusion of 0.14 mg/kg/min dipyridamole combined with four minutes of low-level exercise. Dobutamine stress was used in patients who could not undergo exercise stress or dipyridamole pharmacological stress. At peak stress, 555 MBq ^99m^Tc MIBI was administered, with a similar activity used for the resting imaging.

## Image acquisition and interpretation

Tomographic images were acquired on a dual-headed gamma camera (Siemens ECAM, Erlangen, Germany). A low-energy, high-resolution collimator was used for acquisition with a 140-Kev photopeak and 15% window. A 180° non-circular (body contour) orbit was used.

Images were processed using E soft and 4 DM SPECT processing software (Siemens, Erlangen, Germany) for visual display and quantitative analysis. The summed stress score (SSS), summed rest score (SRS), stress end-systolic volume (SESV) and post-stress left ventricular ejection fraction (SEF) were obtained. The SSS and SRS were categorised into normal, mild, moderate and severe categories using the American College of Cardiology/American Society of Nuclear Cardiology (ACN/ASNC) standard 17-segment model of the left ventricle, and their scoring model and criteria for classification of perfusion.^13^

## Statistical analysis

The data were collected into an excel worksheet and STATA 11 software (Microsoft, Redmond, USA) was used to find any univariate and multivariate correlation between the factors measured. Patients were also divided into two groups depending on whether homocysteine level was raised or not, and compared using a two-tailed paired Students *t*-test with a significance level of *p* < 0.05.

Non-parametric data between those with and without raised homocysteine levels were compared using a Chi-square test. The plasma homocysteine level was also log transformed and correlated with the SSS and SRS and compared with different sub-groups derived from the collected data.

## Results

Of the 120 patients enrolled in the study, 63 (53%) were male. The mean and median age was 56 years (Table 1). Elevated plasma homocysteine levels (> 12 μmol/l) were present in 39 patients (33%). In addition, 38 (32%) patients had diabetes mellitus, 94 (78%) had a history of hypertension, 59 (49%) had dyslipidaemia and 20 (17%) had a significant history of smoking. The group studied was representative of the patient demographics of patients who are referred for suspected myocardial ischaemia at the Steve Biko Academic Hospital, Pretoria.

**Table 1.** Demography And Risk Factors For Patients With A Correlation Between Summed Stress Score (SSS) And Summed Rest Score (SRS) And Elevated Homocysteine Levels

			*Correlation of log homocysteine levels and SSS*		*Correlation of log homocysteine levels and SRS*	
*Demography*	n	*%*	r	p	r	p
Male	63	53	–0.028	ns	0.059	ns
Female	57	47	0.029	ns	–0.003	ns
Age (years)						
26–49	30	25	0.004	ns	0.120	ns
50–64	60	50	0.279	0.03	0.135	ns
65–84	30	25	–0.246	ns	0.014	ns
Racial origin						
Caucasian	77	64	–0.02	ns	–0.022	ns
Coloured	23	19	0.308	ns	0.373	ns
African	20	17	–0.022	ns	–0.082	ns
	*Patients with raised homocysteine*		*Correlation of log homocysteine levels and SSS*		*Correlation of log homocysteine levels and SRS*	
*CAD risk factors*	*n*	*%*	*r*	*p*	*r*	*p*
Diabetes mellitus	38	32	0.055	ns	0.092	ns
Hypertension	94	78	0.155	ns	0.119	ns
Smoker	20	17	0.278	ns	0.073	ns
Dyslipidaemia	59	50	0.120	ns	0.085	ns
Age and gender	88	73	0.133	ns	0.097	ns
Total	120	100	0.077	ns	0.096	ns

CAD = coronary artery disease, *r* = correlation coefficient, *p* = significance, ns = not significant.

Using the criteria of gender and age to determine which patients were considered to be at higher risk for CAD, i.e. males older than 45 years and females older than 55 years, 88 (73%) patients were considered to be at higher risk for CAD. However, there was no effect of the patient’s age on normal or elevated homocysteine levels (Table 1).

The presence of any single individual risk factor did not have a good correlation with plasma homocysteine levels. However the number of risk factors for CAD in an individual patient demonstrated a significant correlation with a raised homocysteine level (*p* = 0.038), such that when four risk factors were present, 61% of patients had an abnormal homocysteine level (Table 2).

**Table 2.** Number Of Risk Factors Per Patient Showing A Significant Correlation Between The Number Of Risk Factors Per Patient And Raised Homocysteine Levels

*Number of risk factors for CAD present in each*	*Patients with the number of risk factors in column 1*	*Patients with raised homocysteine*	
		n	*%*
1	29	5	14
2	24	8	33
3	48	17	35
4	18	11	61

CAD = coronary artery disease, *p* = 0.028 (χ^2^).

Those patients with a raised homocystiene level had a significantly higher SSS and SESV compared to those with a normal homocytsiene level (Table 3). The SEF was also significantly lower in those patients with a raised homocysteine level.

**Table 3.** Mean Myocardial Perfusion Indices In Patients With Elevated And Normal Homocysteine Levels

*Index of myocardial function*	*Elevated homocysteine level*	*Normal homocysteine level*	p
SSS	11.3	6.9	0.02
SRS	3.4	2.1	ns
SEF (%)	54	64	0.02
SESV (ml)	137	105	0.03

SSS = summed stress score, SRS = summed rest score, SEF = post-stress left ventricular ejection fraction, SESV = stress end-systolic volume.

When multivariate regression analysis was applied to all the variables, including risk factors for CAD and SPECT myocardial perfusion indices, only age emerged as a significant factor (Table 4). In addition, univariate analysis showed that patients with elevated homocysteine levels had a lower BMI (26.6 kg/m^2^) compared to those with normal homocysteine levels (30.6 kg/m^2^) (*p* = 0.002).

**Table 4.** Multivariate Regression Analysis For Risk Factors And Myocardial Perfusion Indices And Plasma Homocysteine Levels

*Factor*	*Odds ratio*	*SE*	*Z*	*p > [Z]*
Age	1.076	0.033	2.36	0.019
Diabetes mellitus	1.104	0.681	0.16	ns
Hypertension	3.635	3.280	1.43	ns
Dyslipidaemia	1.209	0.705	0.33	ns
Smoker	3.525	2.771	1.52	ns
SSS	1.002	0.036	0.05	ns
SRS	1.140	0.085	1.75	ns
SEF	0.969	0.029	–1.05	ns
SESV	0.999	0.021	–0.06	ns

SSS = summed stress score, SRS = summed rest score, SEF = post-stress left ventricular ejection fraction, SESV = stress end systolic volume, ns = not significant.

When homocysteine levels were log transformed and the correlation was determined with the myocardial indices, no significant correlation was found for SSS, SRS, SESV and SEF over the whole population, and sub-group analysis found no correlation with various known risk factors (Table 4). We did note however, a significant correlation between homocysteine level and SSS in the patients between 50 and 64 years.

**Fig. 1.**
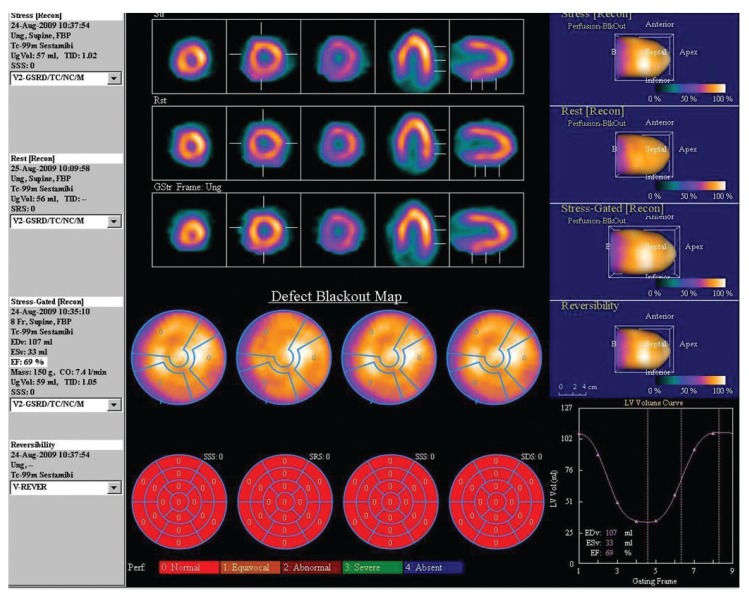
Myocardial perfusion scintigraphy and ‘bullseye’ plots of a 48-year-old male with diabetes, hypertension, dyslipidaemia and significant smoking history. His plasma homocysteine level was normal (7 μmol/l). There was normal perfusion at stress and rest, and the left ventricular ejection fraction was normal (69%).

**Fig. 2.**
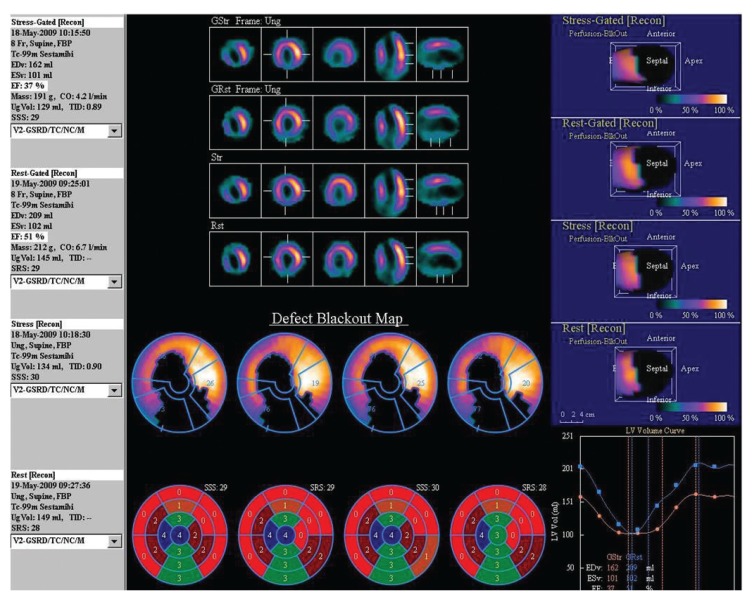
Myocardial perfusion scintigraphy and ‘bullseye’ plots of a 49-year-old male with diabetes, hypertension and significant smoking history. His plasma homocysteine level was elevated (13 μmol/l). ^99m^Tc MIBI imaging at stress and rest showed a significant persistent defect in the anterior wall, apex and inferior wall, with no concurrent ischaemia. The left ventricular ejection fraction was reduced (37%).

The extent of disease was important. A significant correlation was noted between the extent of disease measured by the number of involved coronary artery territories demonstrating infarct, as seen by the SRS evaluated from the myocardial perfusion scintigraphy, and the plasma homocysteine level, where the presence of disease in two or three coronary artery territories was significant (Table 5, Figs 1, 2). Such a correlation was not as strong when comparing the extent of ischaemia as seen by the SSS, with a raised homocysteine level, where there was only a significant correlation in single-vessel disease.

**Table 5.** Correlation Between Elevated Homocysteine And The SSS And SRS In Patients With A Given Number Of Diseased Coronary Artery Territories, As Seen On MPS

*Number of abnormal coronary artery territories*	*SSS*		*SRS*	
	r	p	r	p
0	–0.05	ns	–0.1	ns
1	0.31	0.005	0.1	ns
2	–0.05	ns	0.18	0.049
3	0.15	ns	0.28	< 0.001

SSS = summed stress score, SRS = summed rest score, *r* = correlation co-efficient, *p* = significance level, ns = not significant.

## Discussion

This study examined the relationship between plasma homocysteine levels and myocardial SPECT perfusion indices. Previous investigators have reported a good correlation between scintigraphically detected ischaemia and plasma homocysteine levels in patients on haemodialysis.^14^

A further study demonstrated elevated homocysteine levels with reduced regional left ventricular ejection fraction in an asymptomatic group of young patients,^1^ while others found a high prevalence of patients with elevated homocysteine levels in patients with heart failure with preserved ejection fraction.^15^ This was similar to the figures we obtained where the presence of infarct seemed to have more influence on homocysteine levels than ischaemia.

Another study demonstrated the association of homocysteine with left ventricular dysfunction independent of the presence of CAD. The same study did not demonstrate a significant correlation with myocardial ischaemia.^16^ These results, together with the failure of homocysteine-lowering therapy to reduce cardiac events, show the relationship between raised homocysteine levels and myocardial damage remains poorly understood,^17^-^19^ although there is clearly a need to investigate this link.^20^,^21^

Myocardial SPECT imaging analysed with software such as 4D gives semi-quantitative indices for assessment of myocardial perfusion, in addition to its ability to assess left ventricular function^22^,^23^ and regional wall abnormalities.^24^ When we subdivided our study patients based on the number of affected coronary artery territories, we noted a significant positive but weak correlation between SSS and patients with dual- or triple-vessel CAD, but a stronger link with the SRS, again suggesting infarct was more important than ischaemia. In addition to this finding, we also demonstrated a significant positive correlation between SRS and plasma homocysteine level in patients with single-vessel CAD.

These findings suggest that higher homocysteine levels are associated with more severe and extensive infarct. This may help to explain why such patients with established, irreversible disease are unlikely to benefit from reduction in homocysteine levels alone. This is somewhat confirmed by the findings in our study that higher SESV and lower SEF were observed in patients with elevated homocysteine levels compared to those with normal homocysteine levels. This again suggests that higher homocysteine levels are seen in patients with established and more extensive disease, often resulting in a dilated left ventricle and reduced left ventricular pump efficacy. This correlation between plasma homocysteine level and poorer left ventricular function has been noted in other studies.^1^

The lack of the statistical significance with multivariate regression analysis of other indices, except age, suggests that elevated plasma homocysteine level may well be an independent risk factor for the presence of CAD, as noted in earlier studies.^25^,^26^ We did however demonstrate a significant relationship between the number of risk factors for CAD present in a patient and the presence of elevated plasma homocysteine level.

Most of the patients involved in our study had relatively well-controlled diabetes, hypertension and dyslipidaemia. Despite good control in the study group for these risk factors, there was a higher percentage of patients with increased plasma homocysteine levels among those patients with higher numbers of risk factors for CAD (Table 2). This finding is consistent with several studies^27^,^28^ that show an association of diabetes, hypertension and smoking with plasma homocysteine levels.

This would suggest that despite the association of plasma homocysteine level with smoking, diabetes and hypertension, control of these risk factors does not necessarily result in reduced homocysteine levels. The treatment of underlying risk factors may therefore not provide any benefit to the added risk for CAD, due to the elevated plasma homocysteine levels. These facts may further explain the failure of homocysteine-lowering therapy to reduce cardiac events, as these treatments may be given too late and the raised homocysteine level is less a cause than a marker of established disease.

However, several mechanisms have been proposed for the adverse effects of homocysteine on the endothelium of blood vessels, including coronary arteries. These effects include: decreased bioavailability of nitric oxide, homocysteine-induced oxidative stress, and vascular smooth muscle proliferation, among others.^27^-^30^ Most of these mechanisms also underlie the mechanism by which other risk factors such as diabetes cause endothelial dysfunction.

It may also be postulated that homocysteine initiates the endothelial dysfunction that underlies the CAD, and that once initiated, it is self-propagating, again explaining the link between plasma homocysteine level and the degree of established heart disease, especially as seen with myocardial perfusion scintigraphy.^29^,^31^ This would confirm that lowering homocysteine levels at this late stage in the disease process would offer no benefit.

It has also recently been demonstrated using SPECT myocardial imaging that disease duration and type of therapy provide independent and incremental prognostic information for coronary artery disease in patients with diabetes.^29^,^31^ Patients with elevated homocysteine levels had significantly lower BMI with no significant correlation between homocysteine level and BMI. The lack of significant association was similar to the findings by some groups,^32^ although other groups found at least a weak correlation.^33^,^34^

The statistical significance (*p* > 0.038) for age in the multivariate regression analysis was also noted by previous investigators.^31^ This was probably due to the fact that modifiable risk factors for coronary artery disease, such as diabetes mellitus, hypertension and dyslipidaemia increase with age. Therefore, although age was a risk factor, it was not an independent risk factor for CAD. Finally, the prevalence of elevated homocysteine levels in our study population was similar to previous work done on the prevalence in angiographically proven patients with coronary artery disease in Pretoria, South Africa,^35^ showing there was no significant bias in the patient selection for this study.

A limitation to the study was the reliance on semi-automated analysis of the perfusion indices and LVEF without angiographic correlation. However the advantage of using such a method is the reduction in subjective influence by the operator.

## Conclusion

There was a correlation between plasma homocysteine level and myocardial perfusion indices for patients with scintigraphically proven CAD but not in those patients without disease. We also confirmed that homocysteine level may be an independent risk factor for the presence of CAD, marking the extent of established disease and a higher number of known risk factors. These findings may help to explain why lowering high homocysteine levels is clinically ineffective.
